# Molecular Epidemiology of Emerging Carbapenem Resistance in *Acinetobacter nosocomialis* and *Acinetobacter pittii* in Taiwan, 2010 to 2014

**DOI:** 10.1128/AAC.02007-18

**Published:** 2019-03-27

**Authors:** Feng-Jui Chen, Wei-Cheng Huang, Yu-Chieh Liao, Hui-Ying Wang, Jui-Fen Lai, Shu-Chen Kuo, Tsai-Ling Lauderdale, Huey-Kang Sytwu

**Affiliations:** a National Institute of Infectious Diseases and Vaccinology, National Health Research Institutes, Zhunan, Taiwan; b Institute of Population Health Sciences, National Health Research Institutes, Zhunan, Taiwan; c Department of Microbiology and Immunology, Graduate Institute of Life Sciences, National Defense Medical Center, Taipei, Taiwan

**Keywords:** *Acinetobacter nosocomialis*, *Acinetobacter pittii*, carbapenem resistance, mechanism

## Abstract

This study investigated the molecular epidemiology of carbapenem-resistant Acinetobacter nosocomialis and Acinetobacter pittii (ANAP). Clinical isolates of *Acinetobacter* spp. collected by the biennial nationwide Taiwan Surveillance of Antimicrobial Resistance program from 2010 to 2014 were subjected to species identification, antimicrobial susceptibility testing, and PCR for detection of carbapenemase genes. Whole-genome sequencing or PCR mapping was performed to study the genetic surroundings of the carbapenemase genes. Among 1,041 *Acinetobacter* isolates, the proportion of ANAP increased from 11% in 2010 to 22% in 2014. The rate of carbapenem resistance in these isolates increased from 7.5% (3/40) to 22% (14/64), with a concomitant increase in their resistance to other antibiotics. The *bla*_OXA-72_ and *bla*_OXA-58_ genes were highly prevalent in carbapenem-resistant ANAP. Various genetic structures were found upstream of *bla*_OXA-58_ in different plasmids. Among the plasmids found to contain *bla*_OXA-72_ flanked by XerC/XerD, pAB-NCGM253-like was identified in 8 of 10 isolates. Conjugations of plasmids carrying *bla*_OXA-72_ or *bla*_OXA-58_ to A. baumannii were successful. In addition, three isolates with chromosome-located *bla*_OXA-23_ embedded in AbGRI1-type structure with disruption of genes other than *comM* were detected. Two highly similar plasmids carrying class I integron containing *bla*_IMP-1_ and aminoglycoside resistance genes were also found. The universal presence of *bla*_OXA-272/213-like_ on A. pittii chromosomes and their lack of contribution to carbapenem resistance indicate its potential to be a marker for species identification. The increase of ANAP, along with their diverse mechanisms of carbapenem resistance, may herald their further spread and warrants close monitoring.

## Abstract (precis)

This study investigated the molecular epidemiology of carbapenem-resistant Acinetobacter nosocomialis and Acinetobacter pittii (ANAP). Clinical isolates of *Acinetobacter* spp.

## INTRODUCTION

The Acinetobacter calcoaceticus-Acinetobacter baumannii (Acb) complex has emerged as an important nosocomial pathogen worldwide due to an increasing prevalence in intensive care units, rapid acquisition of various mechanisms of antimicrobial resistance, and association with poor patient outcomes. Among the phenotypically undifferentiated species in the Acb complex, A. baumannii, A. nosocomialis, and A. pittii are clinically relevant but differ in their resistance profiles, virulence, and pathogenicity (1).

Increasing carbapenem resistance in Acb complex, especially in A. baumannii, poses an enormous threat to health care costs and patient outcomes. The main mechanism of carbapenem resistance in the Acb complex is production of carbapenem-hydrolyzing class D β-lactamase (CHDL) and/or metallo-β-lactamase (MBL) (1, 2). Many cross-sectional and longitudinal epidemiological studies have revealed comprehensive portraits of the mechanism and evolution of resistance in A. baumannii (2–6). The most common carbapenemase genes in A. baumannii are *bla*_OXA-23-like_, *bla*_OXA-24-like_, and IS*Aba1-bla*_OXA-51-like_. With the decreasing prevalence of IS*Aba1-bla*_OXA-51-like_, the successful evolution of *Acinetobacter* transposons carrying *bla*_OXA-23-like_ has lead to its worldwide spread (7), and the presence of *bla*_OXA-24-like_ in different plasmids via XerC/XerD module (3) is associated with the high prevalence of *bla*_OXA-24-like_ in certain areas.

A. baumannii has been the focus of most studies, and relatively few studies have been conducted on A. nosocomialis and A. pittii, perhaps due to their low prevalence and low rates of resistance in the past few decades. However, there is currently more interest in A. nosocomialis and A. pittii due to increases in carbapenem resistance and changes in resistance mechanisms (8–13). Previously, OXA-58-like and MBL were primarily responsible for carbapenem resistance in A. nosocomialis and A. pittii, but *bla*_OXA-23-like_ and *bla*_OXA-24-like_ have recently become more common in carbapenem-resistant A. nosocomialis and A. pittii (8–13). The flanking regions and mobile elements surrounding the *bla*_OXA-23-like_ and *bla*_OXA-24-like_ carbapenemase genes are similar to those found in A. baumannii.

Many studies have investigated the clinical impacts and resistance mechanisms of A. nosocomialis and A. pittii (8–13), but there are only few epidemiological studies. The Taiwan Surveillance of Antimicrobial Resistance (TSAR), a longitudinal multicenter surveillance program, collects clinical isolates in Taiwan and tests their susceptibility biennially. In this nationwide study, we used isolates from the 2010 to 2014 TSAR collection to analyze the prevalence, antibiograms, mechanisms of dissemination and resistance, and molecular epidemiology of carbapenem resistance in A. nosocomialis and A. pittii.

## RESULTS AND DISCUSSION

**FIG 1:**
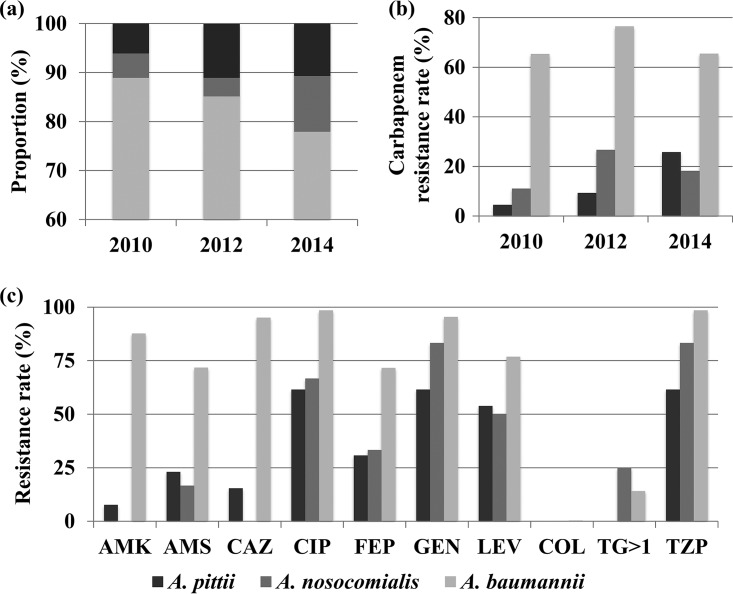
Species distribution and antimicrobial susceptibilities of A. baumannii, A. nosocomialis, and A. pittii from the 2010 to 2014 TSAR collection. The percentages of each *Acinetobacter* spp. based on *gyrB* typing (a), carbapenem resistance in each *Acinetobacter* species (b), and resistance to other antibiotics in carbapenem-resistant A. nosocomialis and A. pittii (c) are shown. AMK, amikacin; AMS, ampicillin/sulbactam; CAZ, ceftazidime; CIP, ciprofloxacin; FEP, cefepime; GEN, gentamicin; LEV, levofloxacin; COL, colistin; TG>1, tigecycline MIC > 1 μg/ml; TZP, piperacillin-tazobactam.

A total of 1,041 Acb complex isolates were collected between 2010 and 2014. The percentages of A. baumannii, A. nosocomialis, and A. pittii determined by *gyrB* typing, and their antibiograms are shown in Fig. 1a and Tables S1 and S2. A. calcoaceticus was not detected from these isolates. Between 2010 and 2014, the percentage of A. baumannii decreased by more than 11%, whereas the percentages of A. nosocomialis and A. pittii increased by 6.4 and 4.6%, respectively (Fig. 1a) and were frequently identified in medical centers in Northern Taiwan (Table S3). Like A. baumannii, hospitalized elderly and adult patients were more commonly affected by A. nosocomialis and A. pittii. A. baumannii was primarily recovered from respiratory specimens, whereas A. nosocomialis and A. pittii were recovered equally from blood and respiratory specimens.

The distribution of A. baumannii, A. nosocomialis, and A. pittii varied among Acb complex isolates from clinical samples in different geographic regions. A. baumannii has been the most prevalent species in Asian countries (14), United Kingdom (15), and the United States (16), ranging from 53 to 79%. However, A. nosocomialis was reported to be more prevalent (46.9%) in Norway, and A. pittii comprised around 60% of the Acb complex in Ireland and Germany (17, 18). Our data indicated that although A. baumannii remained the predominant Acb complex species in Taiwan, its percentage has been decreasing.

In contrast to the persistent high rates of carbapenem resistance in A. baumannii, A. nosocomialis, and A. pittii had been generally more susceptible to carbapenems. The present study found that in 2010, 2012, and 2014, the rates of resistance to carbapenems (imipenem and meropenem) remained stable at 65.4, 76.5, and 65.5% in A. baumannii, but increased from 4.5 to 9.3% to 25.8% in A. pittii and from 11.1 to 26.7% to 18.2% in A. nosocomialis, respectively (Fig. 1b). Carbapenem resistance rates of up to 22% in A. pittii and 53% in A. nosocomialis have been reported in Ireland (17) and South Korea (19). Similar to carbapenem-resistant A. baumannii isolates, which are resistant to multiple antibiotics, our 25 carbapenem-resistant A. nosocomialis and A. pittii isolates also had high rates of resistance to fluoroquinolones, piperacillin-tazobactam, and gentamicin (Fig. 1c).

**FIG 2:**
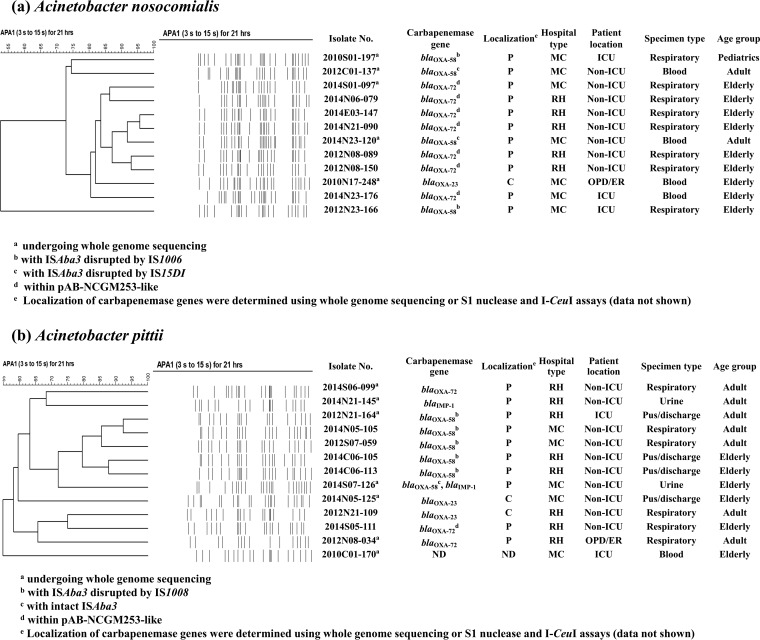
Patient and molecular characteristics of carbapenem-resistant A. nosocomialis (a) and A. pittii (b) in Taiwan. PFGE dendrograms are shown along with carbapenemase genes and their localization (P, plasmid; C, chromosome) and patient characteristics. MC, medical center; RH, regional hospital; ICU, intensive care unit; OPD/ER, outpatient department or emergency room; ND, not detected.

Pulsed-field gel electrophoresis (PFGE) dendrograms of the carbapenem-resistant A. nosocomialis and A. pittii isolates and their carbapenemase genes are shown in Fig. 2. Based on PFGE, the A. nosocomialis isolates from different hospitals were more closely related, whereas the A. pittii isolates were more diverse. PCR and subsequent sequencing showed that the most common carbapenemase genes were *bla*_OXA-58_ in A. pittii and *bla*_OXA-72_ in A. nosocomialis. To delineate their resistance mechanisms and genetic backgrounds, 12 isolates were chosen based on species, pulsotype, and type of carbapenemase genes for whole-genome sequencing (WGS) analysis.

### Plasmid-borne *bla*_OXA-72_ flanked by XerC/XerD-like binding sites.

**FIG 3:**
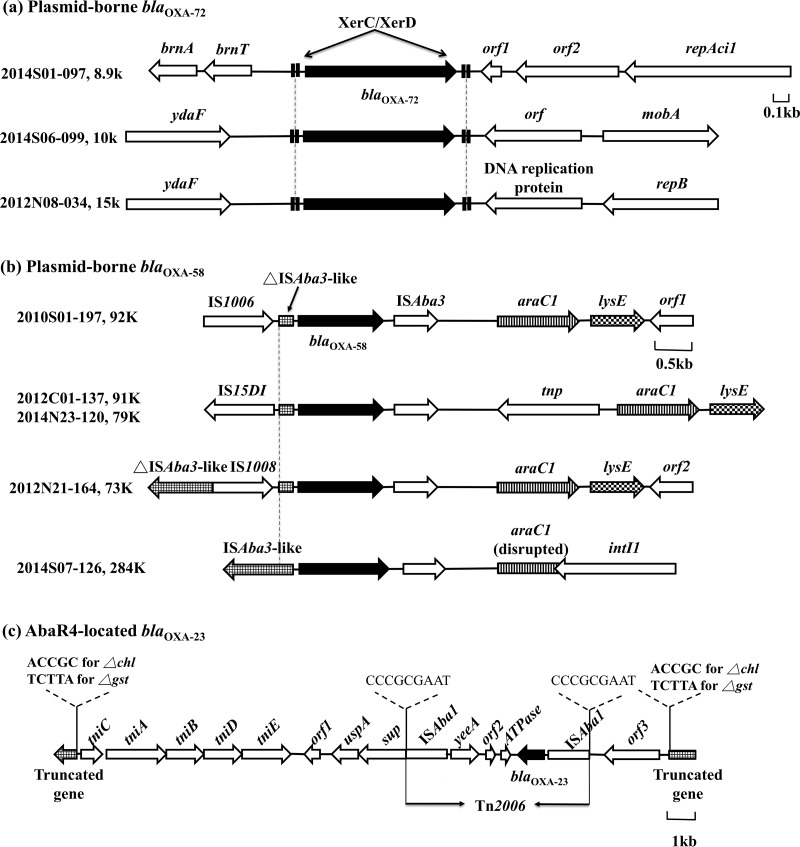
Genetic backgrounds of *bla*_OXA-72_ (a), *bla*_OXA-58_ (b), and *bla*_OXA-23_ (c) in carbapenem-resistant A. nosocomialis and/or A. pittii. The number designations of the isolates sent for WGS and the sizes of the plasmids carrying the carbapenemase genes are listed.

Plasmid pAB-NCGM253, which carries *bla*_OXA-72_, was recently identified in carbapenem-resistant A. nosocomialis isolates in Taiwan (20). PCR mapping showed that all seven A. nosocomialis isolates and one of the three A. pittii isolates with *bla*_OXA-72_ in our collection harbored pAB-NCGM253-like. WGS performed on one of the seven A. nosocomialis (2014S01-097) isolates confirmed the presence of the 8.9-kb pAB-NCGM253 plasmid (accession number AB823544, 100% coverage, 99% identity, Fig. 3a). In addition, WGS confirmed the presence of pAB-NCGM253 in a randomly selected *bla*_OXA-72_-positive A. baumannii isolate (2008S11-069) from our previous collection (21). Transfer of the pAB-NCGM253 plasmid from A. nosocomialis to A. baumannii increased the imipenem MICs of transconjugants by >8-fold (Table 1 ).

**TABLE 1:** Contribution of different resistance determinants from A. nosocomialis and A. pittii to carbapenem resistance in A. baumannii

Strain^*a*^	Original strain	MIC (μg/ml)^*b*^	
		Imipenem	Meropenem
2010N07-100		1	0.5
2010N07-100 (pAB-NCGM253-like with *bla*_OXA-72_)	2014E03-147	>16	>16
2010N07-100 (plasmid with IS*1006*-ΔIS*Aba3-bla*_OXA-58_)	2010S01-197	>16	>16
2010N07-100 (plasmid with IS*15DI*-ΔIS*Aba3-bla*_OXA-58_)	2014N23-120	>16	>16
ATCC 17978 (pYMAb2)		≤0.25	≤0.25
ATCC 17978 (pYMAb2::*bla*_OXA-500_)^*c*^	2014S07-126	≤0.25	≤0.25

a A carbapenem-susceptible A. baumannii clinical isolate (2010N07-100) was the recipient.

b The MICs were determined using the broth microdilution method.

c *bla*_OXA-500_ belongs to the *bla*_OXA-272-like_ branch of *bla*_OXA-213-like_ in A. pittii.

WGS on two A. pittii isolates (2014S06-099 and 2012N08-034) showed that one harbored a 10-kb and the other a 15-kb *bla*_OXA-72_-containing plasmid, neither of which has been previously identified. Conjugation experiments with both plasmids performed on three separate days failed, which may explain their low prevalence. The 10-kb, 15-kb, and pAB-NCGM253 plasmids all contain similar XerC/XerD-like binding sites flanking *bla*_OXA-72_ (Fig. 3a). The XerC/XerD-like binding site has been shown to be involved in the mobilization of *bla*_OXA-72_ from various plasmids in *Acinetobacter* spp. (3, 12, 13).

### Plasmid-borne *bla*_OXA-58_ with different upstream insertion sequences.

WGS performed on three of the four *bla*_OXA-58_-positive A. nosocomialis isolates (2010S01-197, 2012C01-137, and 2014N23-120) and two of the six *bla*_OXA-58_-positive A. pittii isolates (2012N21-164 and 2014S07-126) revealed that *bla*_OXA-58_ exists within different genetic backgrounds (Fig. 3b) and is carried by various plasmids (Table S4). These results indicated a successful and diverse evolutionary history of *bla*_OXA-58_ in the non-*baumannii* Acb complex and its efficient dissemination by plasmids.

A PCR scheme was then designed to detect the upstream genetic structures of *bla*_OXA-58_ in the isolates on which WGS was not performed (Table S5). In all of the four A. nosocomialis isolates containing *bla*_OXA-58_, it was found that the preceding IS*Aba3* is disrupted by an additional insertion sequence. IS*1006* was identified in 2010S01-197 by WGS and in 2012N23-166 by PCR and has been shown to create hybrid promoters for *bla*_OXA-58_ that enhance carbapenem resistance (22). Another insertion sequence, IS*15DI*, in 2012C01-137 and 2014N23-120 also provided an extra promoter, TTTGCA, in addition to TTTATA in IS*Aba3*. In A. pittii, WGS of 2012N21-164 and PCR mapping showed that IS*Aba3* was disrupted by IS*1008* in five of the six isolates containing *bla*_OXA-58_. In the remaining isolate, 2014S07-126, IS*Aba3-bla*_OXA-58_ was intact and, since IS*Aba3-bla*_OXA-58_ has been shown to confer only moderate resistance to carbapenems (22), the carbapenem resistance in 2014S07-126 may come primarily from the *bla*_IMP-1_ gene carried in the same plasmid (see below). Successful transfer of the plasmids carrying IS*1006*- or IS*15DI*-disrupted IS*Aba3-bla*_OXA-58_ in A. nosocomialis to A. baumannii by conjugation revealed their comparable abilities to confer carbapenem resistance (Table 1). The plasmids carrying IS*1008* failed to transfer by conjugation after three independent attempts.

### AbaR4-located *bla*_OXA-23_.

Only one A. nosocomialis isolate and two A. pittii isolates were positive for *bla*_OXA-23,_ and WGS was performed on the A. nosocomialis isolate (2010N17-248) and one of the A. pittii (2014N05-125). In 2010N17-248, the *bla*_OXA-23_ gene was carried on Tn*2006* that was embedded in AbaR4, which is an AbGRI1-type genetic structure (7) (Fig. 3c). The AbaR4 interrupted a gene coding for magnesium chelatase (*chl*) with flanking direct repeats (ACCGC). This type of AbaR4 is prevalent in A. baumannii in Asia (4, 5) and has been found in A. nosocomialis in Korea and Thailand (8, 9).

Two copies of *bla*_OXA-23_ were detected 1.23 Mb apart in 2014N05-125, and both were carried in the same AbaR4 structure described above. One copy of *bla*_OXA-23_ interrupted the *chl* gene, and the other interrupted the gene that codes for glutathione *S*-transferase, *gst*, and was flanked by TCTTA direct repeats. PCR mapping showed the same AbaR4 region in the other *bla*_OXA-23_-positive A. pittii (2012N21-109). In A. baumannii, it has been shown that *comM* (ATPase gene) is commonly interrupted by AbaR4, and this interruption has also been found in A. nosocomialis (8). However, PCR targeting *comM* (6) and an *in silico* search showed that *comM* is not present in our three *bla*_OXA-23_-positive isolates.

### Plasmid-borne class I integron containing *bla*_IMP-1_.

**FIG 4:**
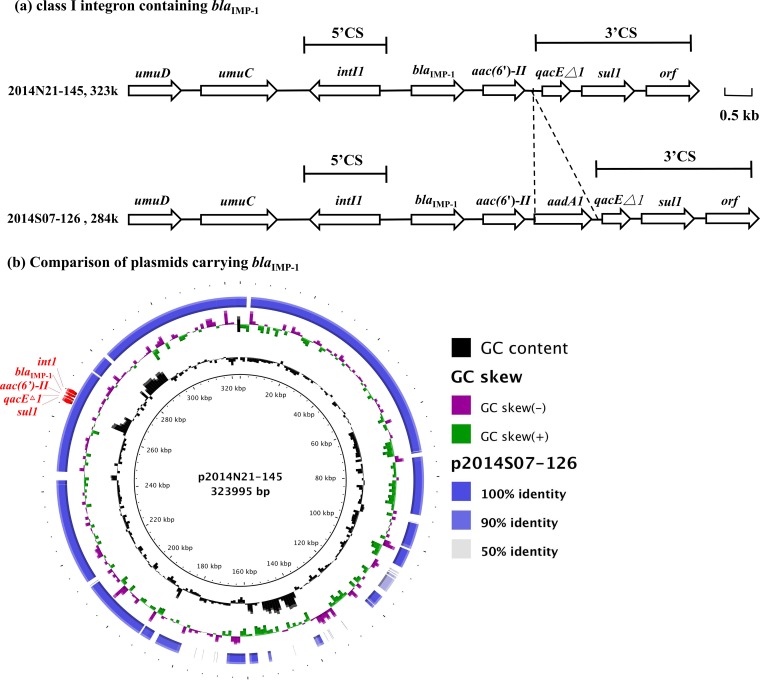
Comparison of flanking regions of *bla*_IMP-1_ (a) and the plasmids carrying *bla*_IMP-1_ (b). A BLAST ring image generator was used to generate an image of the compared plasmids. Plasmid p2014S07-126 was mapped to the reference sequence (p2014N21-145) by BLASTn.

While CHDLs are the most prevalent mechanisms of carbapenem resistance in A. baumannii, MBLs, such as Verona integron-encoded MBL (VIM), imipenemase (IMP), and Seoul imipenemase MBL (SIM), have also been found in *Acinetobacter* spp. In this study, two A. pittii isolates, 2014N21-145 and 2014S07-126, harbored *bla*_IMP-1_ embedded in class I integron. The integron incorporated additional aminoglycoside resistance determinants, *aac(6′)-II* and/or *aadA1* (Fig. 4a). Plasmids p2014N21-145 (323 kb) and p2014S07-126 (284 kb) that contained the integron shared a 256-kb region with 99% similarity (Fig. 4b, accession numbers CP033569 and CP033531). An additional IS*Aba3-bla*_OXA-58_ was located in p2014S07-126 in the region that shared little similarity with p2014N21-145.

The *bla*_IMP-like_ gene was first discovered in Pseudomonas aeruginosa in 1991 and has since been reported in Enterobacteriaceae and *Acinetobacter* spp. (23–25). Although the prevalence of *bla*_IMP-like_ has been relatively low, many variants of *bla*_IMP-like_ have been identified worldwide in clinical *Acinetobacter* isolates (2). Similar to other MBL genes (26), the *bla*_IMP-like_ gene is usually incorporated into class I integron in *Acinetobacter* spp. (25, 27). In this survey, the similarities of plasmids carrying *bla*_IMP-1_, which were identified in isolates from different geographic areas, suggest that plasmids may be responsible for transferring integron containing *bla*_IMP-like_. However, transfer of the p2014N21-145 and p2014S07-126 plasmids in A. pittii to A. baumannii by conjugation failed three independent times.

### High prevalence of chromosomal *bla*_OXA-272-like_ branch of *bla*_OXA-213-like_ in *A. pittii*.

WGS revealed the presence of *bla*_OXA-213-like_ on the chromosomes of A. pittii isolates in the TSAR collection. The amino acid sequences of OXA-213-like in our isolates, OXA-213-like in A. calcoaceticus from the NCBI database, and other common CHDLs were compared (Fig. S1a). The phylogenetic tree showed the difference of OXA-213-like in A. pittii from OXA-213-like in A. calcoaceticus and other common CHDL. Based on NCBI data, the 85 to 90% similarity between the OXA-213-like in our A. pittii and in A. calcoaceticus (Fig. S1b) may not justify the creation of an extra name of OXA. However, to differentiate our OXA-213-like in A. pittii and naturally occurring OXA-213-like in A. calcoaceticus (28), OXA-272-like branch of OXA-213-like, which is the oldest and founding member of the subgroup, is used in this article.

One previous study (29) using *in silico* search suggested *bla*_OXA-272-like_ may be intrinsic to A. pittii. With more clinical isolates tested, our finding supports the suggestion. Since the primer set in the previous study (30) that identified the *bla*_OXA-213-like_ in A. calcoaceticus was unable to identify *bla*_OXA-272-like_ in our study (data not shown), new PCR primers were developed to identify *bla*_OXA-272-like_ and to differentiate them from *bla*_OXA-213-like_ in A. calcoaceticus. This PCR scheme was positive in all of the 13 imipenem-resistant A. pittii isolates in this study and in 13 imipenem-susceptible A. pittii isolates randomly selected from TSAR. Sequencing of the amplicons using extra-genetic primers confirmed them to be *bla*_OXA-272-like_. Furthermore, PCR of randomly selected 13 A. nosocomialis and 13 A. baumannii isolates from TSAR, and two reference strains of A. calcoaceticus (ATCC14987 and ATCC23055) were all negative for *bla*_OXA-272-like_.

Since this study included isolates from 2010 to 2014, it is possible that *bla*_OXA-272-like_ was present in A. baumannii and A. nosocomialis isolates before 2010. Therefore, we collected bacteremic Acb complex isolates from 1997 to 2015 from four different hospitals (Table S6) and tested for carbapenem susceptibility and for carbapenem resistance genes in A. baumannii, A. nosocomialis, and A. pittii (A. calcoaceticus was not identified in the collection). PCR showed that all 20 of the carbapenem-susceptible A. pittii isolates contained *bla*_OXA-272-like_. In contrast, none of the A. baumannii or A. nosocomialis isolates contained *bla*_OXA-272-like_ or *bla*_OXA-213-like_. Transformation of pYMAb2::*bla*_OXA-272-like_ into A. baumannii clinical isolate 2010N07-100 did not confer resistance to imipenem or meropenem (Table 1). Thus, *bla*_OXA-272-like_ may serve as a marker for A. pittii, just like *bla*_OXA-51-like_ is for A. baumannii.

In conclusion, this longitudinal nationwide survey revealed an increase of A. nosocomialis and A. pittii among Acb complex isolates recovered from clinical samples in Taiwan. The prevalence of carbapenem resistance also increased in these two species. Various carbapenem resistance determinants with diverse surrounding genetic structures were identified, with plasmid-borne *bla*_OXA-72_ or *bla*_OXA-58_ being the main mechanisms, but AbaR4-located *bla*_OXA-23_ in the chromosome and plasmid-borne class I integron containing *bla*_IMP-1_ were also detected.

## MATERIALS AND METHODS

### Isolate collection, species identification, and antimicrobial susceptibility testing.

The *Acinetobacter* isolates were identified from the 2010–2014 TSAR collection of clinical isolates (corresponding to TSAR VII to IX). The isolate collection protocol and participating hospitals have been described previously (31). All of the clinical isolates identified initially as Acb complex by Vitek II GN card (bioMérieux, Marcy l’Etoile, France) were further identified to the species level using the *gyrB* PCR typing method (32). MICs were determined by the reference broth microdilution method using Sensititre custom-designed plates. Susceptibility was determined in accordance with the Clinical and Laboratory Standards Institute (CLSI) (33). Carbapenem resistance was defined as MICs of >4 μg/ml for both imipenem and meropenem.

### Detection of carbapenemase genes.

The presence of genes encoding class A (*bla*_NMC_, *bla*_SME_, *bla*_IMI_, *bla*_KPC_, and *bla*_GES_), class B (*bla*_IMP_, *bla*_VIM_, *bla*_GIM_, *bla*_SPM_, *bla*_SIM-1_, and *bla*_NDM-1_), and class D (*bla*_OXA-23-like_, *bla*_OXA-24-like_, *bla*_OXA-58-like_, and *bla*_OXA-51-like_) β-lactamases with carbapenemase activity was detected by PCR in the carbapenem-resistant isolates (34–36). PCR to detect IS*Aba1* upstream of *bla*_OXA-23-like_ or *bla*_OXA-51-like_ was performed using the reverse IS*Aba1* primer and the reverse target gene primer (34–36). All detected carbapenemase genes were sent for sequencing.

### Modified PCR scheme to detect the genetic background upstream of *bla*_OXA-58_.

Due to various forms of IS*Aba3* and/or insertion sequences upstream of *bla*_OXA-58,_ a three-step PCR scheme (Fig. S2 and Table S5) was modified from previous published protocols (22, 37) to determine the presence of *bla*_OXA-58_ and its upstream genetic structure. The first step was to detect for *bla*_OXA-58_. The second step was to detect for truncated or complete IS*Aba3* using primers that targeted the conserved part of IS*Aba3*. The third step was to detect for hybrid promoters upstream of *bla*_OXA-58_. Based on sequencing results from this study and on sequence data in the NCBI database, we determined that the sequences of the hybrid promoters are mostly conserved and have only minor differences (Fig. S2a). Three sets of forward primers targeting hybrid promoters were designed, and a mixture of two of the sets of forward primers successfully identified all of the hybrid promoter variants (Fig. S2b). To further identify the insertion sequences that interrupted IS*Aba3*, an optional PCR using the primer sets listed in Table S5 was performed.

### PCR for the detection of pAB-NCGM253-like and *bla*_OXA-272-like_.

Six sets of primers covering overlapping areas of pAB-NCGM253 and amplification programs are listed in Table S5. Two sets of primers targeting *bla*_OXA-272-like_ and *bla*_OXA-213-like_, respectively, were designed (Table S5). The program included 30 cycles of denaturation at 94°C for 30 s, annealing at 50°C for 30 s, and elongation at 72°C for 30 s.

### PFGE.

All of the carbapenem-resistant isolates were subjected to PFGE after digestion with ApaI as previously described (38). The stained gels were photographed and BioNumerics software (Applied Maths) was used to generate dendrograms to determine the relatedness of the isolates.

### WGS using oxford nanopore technologies.

Isolates were then selected for WGS based on their species, resistance mechanism, and pulsotype. A Nanopore sequencing library was constructed using ligation methodology with a ligation sequencing kit 1D (SQK-LSK108) and a native barcoding kit (EXP-NBD103). Briefly, 10-kb genomic DNA fragments were generated by g-TUBE (Covaris). DNA fragments were repaired and dA tailed with a NEBNext FFPE DNA repair mix and a NEBNext Ultra II End Repair/dA-Tailing module (New England BioLabs). Individual barcodes were added to the dA-tailed DNA using NEB Blunt/TA Ligase Master Mix (New England BioLabs). Equimolar amounts of the barcoded DNAs were pooled and adaptors were attached using NEBNext Quick Ligation module (New England BioLabs). The library was loaded into a SpotON Flowcell R9.4 or 9.5 (FLO-MIN106 or FLO-MIN107), and the sequencing script NC_48Hr_Sequencing_FLO-MIN106 or 107_SQK_LSK108 was executed on MinKNOW (v1.7.14). Albacore (v2.1.7) was used for demultiplexing and base calling. The base-called sequencing reads were then *de novo* assembled by Canu (v1.6) (39) or minimap2 (v2.10)/miniasm (v0.2) (40, 41). The assembled contigs were subsequently polished with the sequencing reads using Nanopolish (v0.8.5) (https://github.com/jts/nanopolish) and Racon (v1.1.1) (42), respectively. Finally, redundant ends of the consensus sequences were trimmed, and the circular sequences were rearranged to begin at DnaA/RepA or a position with the minimum value of GC skew. These processes were all implemented in an in-house pipeline to produce the complete genomes of the sequenced strains.

### Conjugation and transformation experiments.

Conjugation was performed following previously described protocols (43). Briefly, 500 μl of the donor culture in Luria-Bertani (LB) broth and 500 μl of the recipient culture in LB broth were mixed with 400 μl of 10 mM MgSO_4_, and the mixture was distributed evenly onto a filter. The filter was transferred to Mueller-Hinton agar and incubated at 37°C for 16 h. The mated cells were harvested by submerging the filter in 5 ml of LB broth and then vortexing the sample. The suspended bacteria were plated onto selective LB agar containing 2 μg/ml imipenem and another selective antibiotic and then incubated at 37°C overnight. Transconjugants were tested for target genes by PCR and for antibiotic susceptibility by using the Vitek II automated system and broth microdilution.

The *bla*_OXA-272-like_ gene was PCR amplified with a proofreading DNA polymerase (Phusion high-fidelity DNA polymerase; Finnzymes, Espoo, Finland) and cloned into the pCRII-TOPO vector (Invitrogen, Carlsbad, CA). After confirmation of *bla*_OXA-272-like_ by sequencing (Mission Biotech, Taipei, Taiwan), the pCRII-TOPO::*bla*_OXA-272-like_ was digested with XbaI and XhoI, and the digested *bla*_OXA-272-like_ fragment was cloned into the Escherichia coli-A. baumannii shuttle vector pYMAb2. The recombinant plasmid and a control plasmid (pYMAb2 without *bla*_OXA-272-like_) were transformed into kanamycin-susceptible A. baumannii ATCC17978 by electroporation with a gene pulser electroporator (Bio-Rad, Hercules, CA) using 2-mm electrode gap cuvettes (44). The pYMAb2 plasmid contains a kanamycin resistance gene, and kanamycin-resistant transformants were selected. Sequencing was performed to confirm the presence of *bla*_OXA-272-like_.

### Data availability.

The nucleotide sequences of 2010N17-248, 2014N05-125, 2012N08-034, 2014S06-099, 2014S01-097, 2010S01-197, 2012C01-137, 2014N23-120, 2012N21-164, 2014S07-126, 2014N21-145, 2010C01-170, and 2008S11-069 (K069 in a previous study [21]) were deposited in GenBank under the accession numbers CP033572 to CP033573, CP033525 to CP033529, CP033520 to CP033524, CP033540 to CP033544, CP033550 to CP033556, CP033561 to CP033567, CP033557 to CP033560, CP033545 to CP033549, CP033535 to CP033539, CP033530 to CP033534, CP033568 to CP033571, CP029489, and CP033516 to CP033519, respectively.

## Supplementary Material

Supplemental file 1
