# Supplementary material for: Molecular Epidemiology of Emerging Carbapenem Resistance in Acinetobacter nosocomialis and Acinetobacter pittii in Taiwan, 2010 to 2014
Source: Antimicrob Agents Chemother. 2019 Mar 27;63(4):e02007-18. doi: 10.1128/AAC.02007-18 (PMC6496155; doi:10.1128/AAC.02007-18)
Supplement: Supplemental file 1 [file AAC.02007-18-s0001.pdf]

Supplementary Table S1. Antimicrobial resistance in *A. baumannii* from the 2010-2014 TSAR collection

	TSAR VII (2010) N = 321		TSAR VIII (2012) N = 332		TSAR IX (2014) N = 226		Overall N = 879	
	N	%	N	%	N	%	N	%
AMK	226	70.4	246	74.1	149	65.9	621	70.6
AMS	168	52.3	206	62	106	46.9	480	54.6
CAZ	251	78.2	269	81	169	74.8	689	78.4
CIP	265	82.6	280	84.3	180	79.6	725	82.5
FEP	161	50.2	211	63.6	89	39.4	461	52.4
GEN	255	79.4	267	80.4	164	72.6	686	78
IPM	210	65.4	254	76.5	148	65.5	612	69.6
LEV	174	54.2	240	72.3	147	65	561	63.8
MEM	211	65.7	254	76.5	149	65.9	614	69.9
COL	0	0	0	0	2	0.9	2	0.2
TG > 1	7	2.2	68	20.5	22	9.7	97	11
TZP	225	70.1	268	80.7	156	69	649	73.8

[a] AMK, amikacin; AMS, ampicillin/sulbactam; CAZ, ceftazidime; CIP, ciprofloxacin; FEP, cefepime; GEN, gentamicin; IPM, imipenem; LEV, levofloxacin; MEM, meropenem; COL, colistin; TG > 1, tigecycline MIC > 1 mg/L; TZP, piperacillin/tazobactam

Supplementary Table S2. Antimicrobial resistance in *A. nosocomialis* and *A. pittii* from the 2010-2014 TSAR collection

	TSAR VII (2010) N = 40		TSAR VIII (2012) N = 58		TSAR IX (2014) N = 64		Overall N = 162	
	N	%	N	%	N	%	N	%
AMK	1	2.5	1	1.7	2	3.1	4	2.5
AMS	1	2.5	1	1.7	4	6.3	6	3.7
CAZ	1	2.5	0	0	2	3.1	3	1.9
CIP	6	15	12	20.7	16	25	34	21
FEP	2	5	2	3.4	6	9.4	10	6.2
GEN	6	15	12	20.7	19	29.7	37	22.8
IPM	3	7.5	9	15.5	14	21.9	26	16
LEV	3	7.5	9	15.5	13	20.3	25	15.4
MEM	3	7.5	8	13.8	14	21.9	25	15.4
COL	0	0	0	0	1	1.6	1	0.6
TG > 1	0	0	2	3.4	1	1.6	3	1.9
TZP	2	5	7	12.1	16	25	25	15.4

[a] AMK, amikacin; AMS, ampicillin/sulbactam; CAZ, ceftazidime; CIP, ciprofloxacin; FEP, cefepime; GEN, gentamicin; IPM, imipenem; LEV, levofloxacin; MEM, meropenem; COL, colistin; TG > 1, tigecycline MIC > 1 mg/L; TZP, piperacillin/tazobactam

Supplementary Table S3. Comparison of patient characteristics of *A. baumannii* versus *A. nosocomialis* and *A. pittii* from the 2010-2014 TSAR collection

	A. baumannii N = 879		*A. nosocomialis* and *A. pittii* N = 162		
	N	%	N	%	P-value[a]
Hospital type					
Medical centers	364	41.4	94	58.0	< 0.001
Region					< 0.001
North	243	27.7	71	43.8	
Central	370	42.1	35	21.6	
South	184	20.9	43	26.5	
East	82	9.3	13	8.0	
Specimen type					< 0.001
Respiratory	476	54.2	61	37.7	
Blood	131	14.9	68	42.0	
Urine	121	13.8	9	5.6	
Pus/discharge	99	11.3	15	9.3	
Others	52	5.9	9	5.6	
Age[b]					< 0.001
< 18	24	2.7	9	5.6	
18-65	280	31.9	67	41.4	
> 65	571	65.0	86	53.1	
Patient location[b]					< 0.001
OPD/ER	71	8.1	14	8.6	
Non-ICU	469	53.4	118	72.8	
ICU	337	38.3	30	18.5	

[a] Chi-squared test

[b] Information regarding age and patient location was not available for some patients.

Supplementary Table S4. Comparison of plasmids carrying *bla*$_{OXA\text{-}58\text{-}like}$ in this study and in the NCBI database

Isolate No. Accession No.	Plasmid size	Upstream insertion sequences	Accession No. of the best matched plasmid, host species	Plasmid size	Carbapenemase gene in the best matched plasmid	Coverage	Similarity
2010S01-197 CP033563	92 K	IS*1006*-IS*Aba3*	No comparative plasmid	-	-	-	-
2012C01-137 CP033558	91 K	IS*15DI*- IS*Aba3*	CP026086.1, *A. pittii*	112 K	*bla*$_{OXA\text{-}58\text{-}like}$	96%	99%
2014N23-120 CP033547	79 K	IS*15DI*- IS*Aba3*	CP026086.1, *A. pittii*	112 K	*bla*$_{OXA\text{-}58\text{-}like}$	98%	99%
2012N21-164 CP033537	73 K	IS*1008*-IS*Aba3*	LN833432.1, *A. baumannii*	85 K	*bla*$_{NDM\text{-}1}$	67%	98%
2014S07-126 CP033531	284 K	IS*Aba3*	CP010351.1, *A. johnsonii*	399 K	Not detected	87%	99%

Supplementary Table S5. Primers used to detect for *bla*$_{OXA-58-like}$ and its upstream regions, pAB-NCGM253, and *bla*$_{OXA-213/272-like}$

Target	Primers	Sequences	Size (bp)	Reference
bla$_{OXA-58-like}$	OXA-58-like-F	CGATCAGAATGTTCAAGCGC	523	Poirel et al [a]
	OXA-58-like-R	CCCCTCTGCGCTCTACATAC		Woodford et al [b]
IS*Aba3*- *bla*$_{OXA-58-like}$	IS*Aba3*-2F	TATACTATCACTGAGGCAGGTT	773	This study
	OXA-58-like-R	CCCCTCTGCGCTCTACATAC		Woodford et al [b]
Hybrid promoters-*bla*$_{OXA-58-like}$	Hybrid-P$_{C2}$	TTGCAACAGTG**CC**ATTTT	795	This study
	Hybrid-P$_{C3}$	TTGCAACAGTG**CCC**ATTTT		This study
	OXA-58-like-R	CCCCTCTGCGCTCTACATAC		Woodford et al [b]
IS*15DI-bla*$_{OXA-58-like}$	IS15DI-F	TACAGATACGCCCAGCGG	1124	This study
	OXA-58-like-R	CCCCTCTGCGCTCTACATAC		Woodford et al [b]
IS*1006-bla*$_{OXA-58-like}$	IS1006-F	GGAAGGTAAATGTCCACCAGACC	1084	This study
	OXA-58-like-R	CCCCTCTGCGCTCTACATAC		Woodford et al [b]
IS*1008-bla*$_{OXA-58-like}$	IS1008-F	CCTACAGATTTACATTCATGGC	1330	This study
	OXA-58-like-R	CCCCTCTGCGCTCTACATAC		Woodford et al [b]

Exclusively *bla*$_{OXA-272-like}$	AP-OXA272-F	CCATTGGTACGATGT	265	This study
	AP-OXA272-R	TGTTGCTTTATGATGC		This study
bla$_{OXA-213-like}$ not including *bla*$_{OXA-272-like}$	AC-OXA213-F	CGCCCTCTAGAATGAGTATTCAAC	150	This study
	AC-OXA213-R	GTGAAGGATCCTTACCAATGCTTAAT		This study
pAB-NCGM253	OXA24-NCGM1-F	TATGTACTCATGATTGTACA	1980[c]	This study
	OXA24-NCGM1-R	GCTTAATATGACTTTAGCAT		This study
	OXA24-NCGM2-F	CATATTCTGTGTGAGATAGC	1990[c]	This study
	OXA24-NCGM2-R	ATTAAAGTTTTACAGAATAA		This study
	OXA24-NCGM3-F	CGAATAGAACCAGACATTCC	1373[c]	This study
	OXA24-NCGM3-R	AACCATGCTCATATTTGTTTCG		This study
	OXA24-NCGM4-F	TCTAAAATTAACATAATACG	2180[c]	This study
	OXA24-NCGM4-R	GTCACGCCAGTATTAACCAA		This study
	OXA24-NCGM5-F	CGCCCATAAATTACACCTCT	1241[d]	This study
	OXA24-NCGM5-R	AGGCATTGTCCTTTACAACT		This study

	OXA24-NCGM6-F	AAATCATCCGAATCATTAGTGCA	889[e]	This study
	OXA24-NCGM6-R	GACTTAGCTAAACTTGGTTCAAC		This study

[a] Poirel L, Marqué S, Héritier C et al. OXA-58, A Novel Class D β-Lactamase Involved in Resistance to Carbapenems in *Acinetobacter baumannii*. Antimicrob Agents Chemother 2005; **49**: 202–208.

[b] Woodford N, Ellington MJ, Coelho JM et al. Multiplex PCR for genes encoding prevalent OXA carbapenemases in *Acinetobacter* spp. Int J Antimicrob Agents 2006; **27**: 351-3.

[c] 30 cycles of denaturation at 94 °C for 30 s, annealing at 53°C for 30s and elongation at 72 °C for 2.5 min.

[d] 30 cycles of denaturation at 94 °C for 30 s, annealing at 58°C for 30s and elongation at 72 °C for 1.5 min.

[e] 30 cycles of denaturation at 94 °C for 30 s, annealing at 54°C for 30s and elongation at 72 °C for 1 min.

Supplementary Table S6. Detection of *bla*$_{OXA-272-like}$ in *Acinetobacter* spp. by PCR

	A. baumannii (n = 20)		*A. nosocomialis* (n = 20)		*A. pittii* (n = 20)
	CR (n = 10)	CS (n = 10)	CR (n = 10)	CS (n = 10)	CS (n = 20)
No. of isolates from each hospital [a]	1/2/5/2	0/7/1/2	3/4/2/1	3/3/1/3	14/0/0/6
Year	2010-2013	2004-2013	2003-2015	2004-2013	2012-2014
Imipenem MIC range	8-128	0.25-2	8-64	0.125-2	0.25-1
Meropenem MIC range	8-64	0.25-2	8-128	0.25-2	0.25-0.5
Carbapenemase genes [b]					
bla$_{OXA-23-like}$	8	0	1	0	0
bla$_{OXA-24-like}$	2	0	5	0	0
IS*Aba1*- *bla*$_{OXA-51-like}$	2	0	0	0	0
Hybrid promoter-*bla*$_{OXA-58-like}$	1	0	4	0	0
bla$_{OXA-272-like}$	0	0	0	0	20
bla$_{IMP}$	0	0	0	0	0
bla$_{VIM}$	0	0	0	0	0

CR, carbapenem-resistant; CS, carbapenem-susceptible

[a] Hospitals included Tri-Service General Hospital, Taipei Veterans General Hospital, Mackay Memorial Hospital, and Changhua Christian Hospital.

[b] An isolate may contain multiple carbapenemase genes.

Supplementary Figure S1. Dendrogram of amino acid sequences of carbapenem-hydrolyzing class D β-lactamases (CHDL) in *A. calcoaceticus-baumannii* complex isolates and intrinsic CHDLs in other *Acinetobacter* species (a) and comparison of the amino acid sequences of OXA-272-like and OXA-213-like (b). (a) Amino acid sequences of our isolates and amino acid sequences from NCBI were compared. Accession no. (or isolate no. from this study), and CHDL type are indicated. The CHDL in *A. pittii* belonged to OXA-272-like and those in *A. calcoaceticus* belonged to OXA-213-like. The species was identified based on the sequence of *gyr*B. (b) Multiple amino acid sequence alignment was generated using default settings of CLUSTALW at https://npsa-prabi.ibcp.fr/cgi-bin/npsa_automat.pl?page=/NPSA/npsa_server.html.

(a)

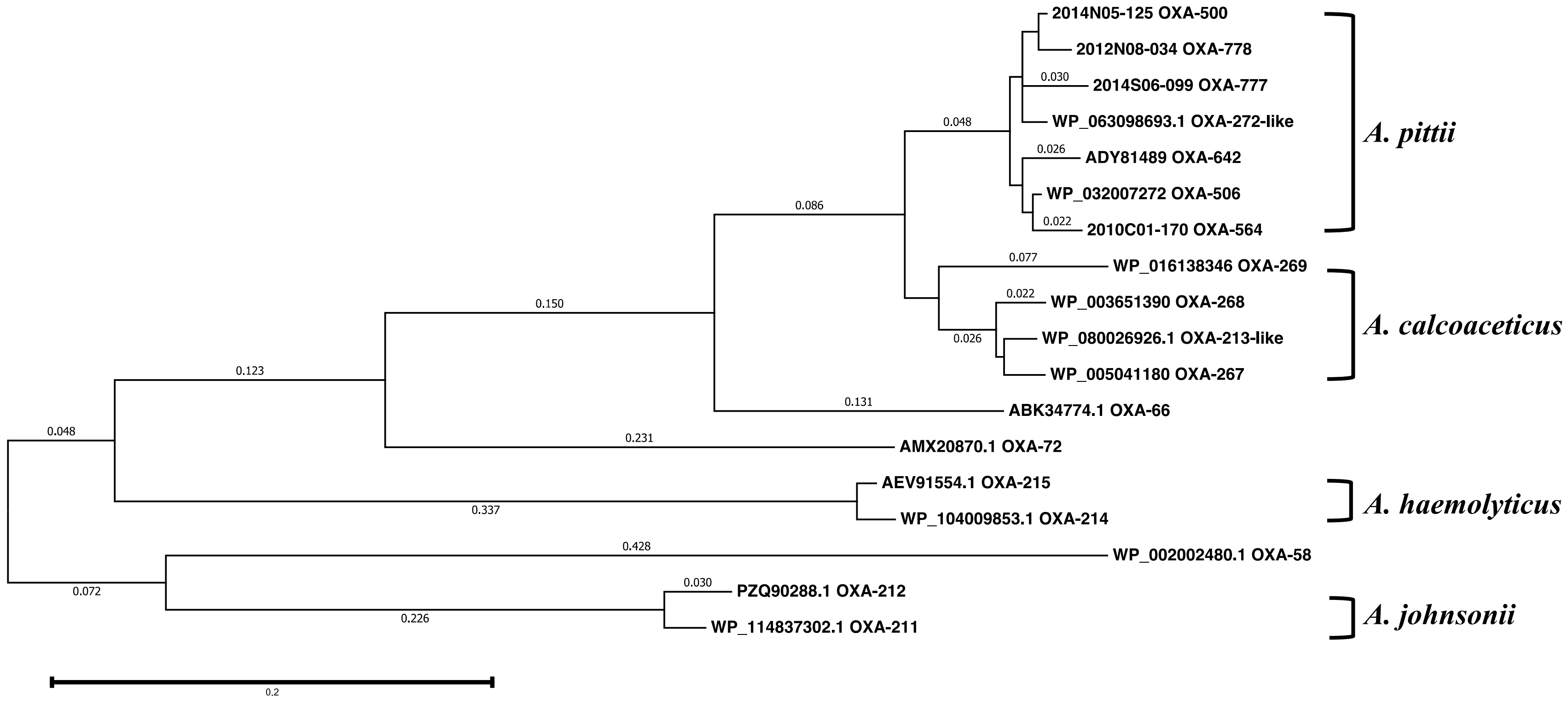
2014N05-125 OXA-500
2012N08-034 OXA-778
2014S06-099 OXA-777
WP_063098693.1 OXA-272-like
ADY81489 OXA-642
WP_032007272 OXA-506
2010C01-170 OXA-564
WP_016138346 OXA-269
WP_003651390 OXA-268
WP_080026926.1 OXA-213-like
WP_005041180 OXA-267
ABK34774.1 OXA-66
AMX20870.1 OXA-72
AEV91554.1 OXA-215
WP_104009853.1 OXA-214
WP_002002480.1 OXA-58
PZQ90288.1 OXA-212
WP_114837302.1 OXA-211
A. pittii
A. calcoaceticus
A. haemolyticus
A. johnsonii
0.030
0.048
0.026
0.022
0.086
0.077
0.022
0.026
0.150
0.123
0.131
0.048
0.231
0.337
0.428
0.072
0.030
0.226
0.2

(b)

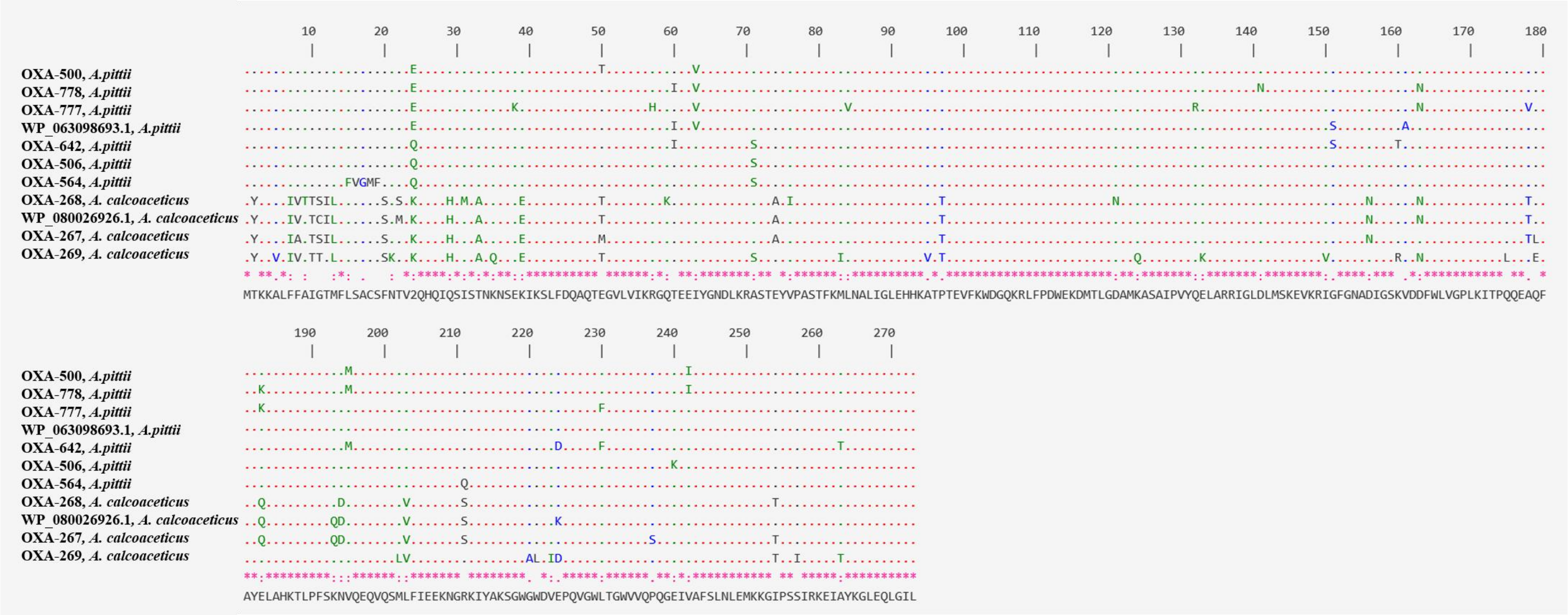
OXA-500, A.pittii
OXA-778, A.pittii
OXA-777, A.pittii
WP_063098693.1, A.pittii
OXA-642, A.pittii
OXA-506, A.pittii
OXA-564, A.pittii
OXA-268, A. calcoaceticus
WP_080026926.1, A. calcoaceticus
OXA-267, A. calcoaceticus
OXA-269, A. calcoaceticus
MTKKALFFAIGTMFLSACSFNTV2QHQIQSISTNKNSEKIKSLFDQAQTEGVLVIKRGQTEEIYGNDLKRASTEYVPASTFKMLNALIGLEHHKATPTEVFKWDGQKRLFPDWEKDMTLGDAMKASAIPVYQELARRIGLDLMSKEVKRIGFGNADIGSKVDDFWLVGPLKITPQQEAQF
AYELAHKTLPFSKNVQEQVQSMLFIEEKNGRKIYAKSGWGWDVEPQVGWLTGWVVQPQGEIVAFSLNLEMKKGIPSSIRKEIAYKGLEQLGIL

Supplementary Figure S2. Scheme to identify the genetic background of *bla*$_{OXA-58}$ (a) and validation of the mixture of forward primers in step 3 (b). The number of cytosines in hybrid promoter regions, which are underlined in (a), differed in our isolates and ranged from one to three in isolates C_2 (2012N21-164), C_3 (2014N23-120), and C_4 (2010S01-197), respectively. Sequences deposited in NCBI also showed this variation (accession no. CP026086.1, JQ241789, and GU327621.1). Therefore, three sets of forward primers (Hybrid-P_{C2}, -P_{C3}, and -P_{C4}) were designed. PCR with the mixture of Hybrid-P_{C2} and Hybrid -P_{C3} primers yielded the best results. M, marker; NC negative control.

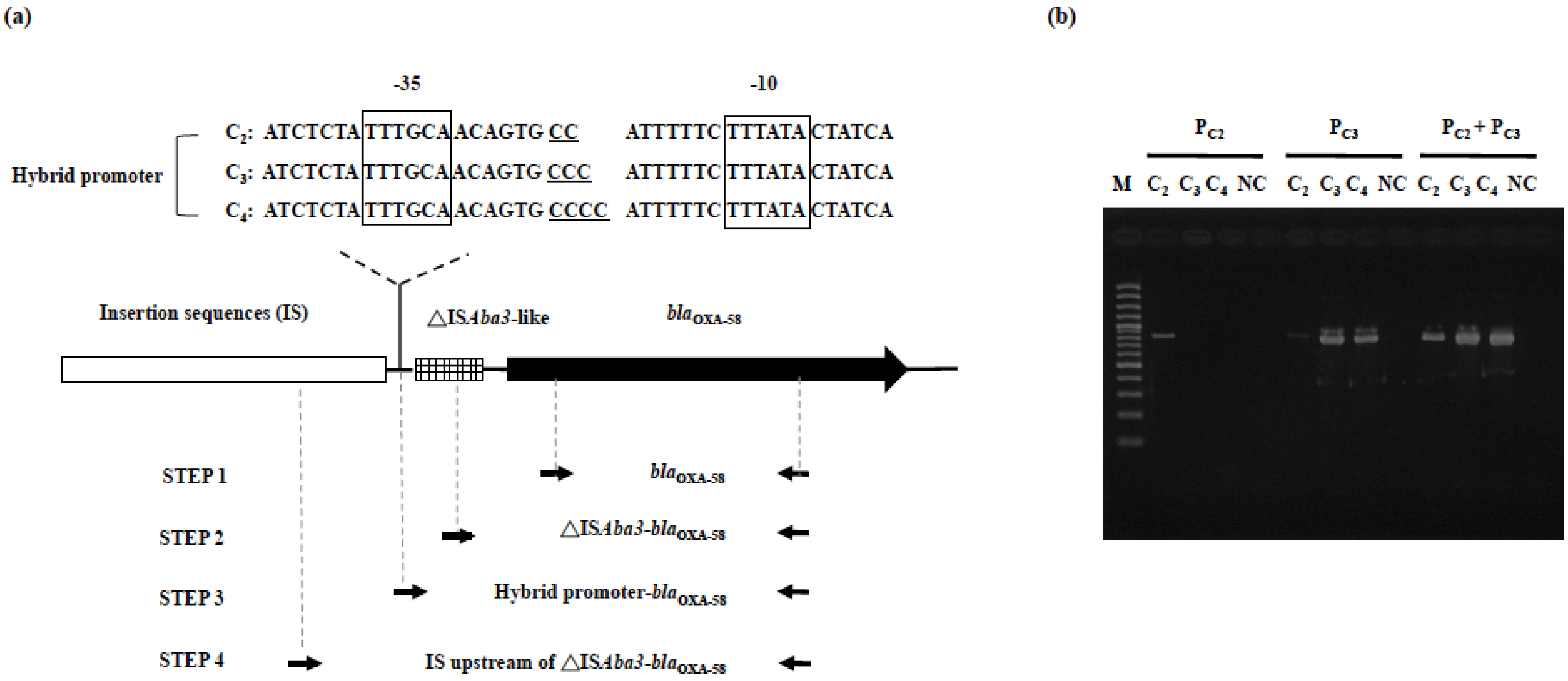

(a)
-35
-10
Hybrid promoter
C2: ATCTCTA TTTGCA ACAGTG CC ATTTTTC TTTATA CTATCA
C3: ATCTCTA TTTGCA ACAGTG CCC ATTTTTC TTTATA CTATCA
C4: ATCTCTA TTTGCA ACAGTG CCCC ATTTTTC TTTATA CTATCA
Insertion sequences (IS)
△ISAba3-like
blaOXA-58
STEP 1
blaOXA-58
STEP 2
△ISAba3-blaOXA-58
STEP 3
Hybrid promoter-blaOXA-58
STEP 4
IS upstream of △ISAba3-blaOXA-58
(b)
PC2
PC3
PC2 + PC3
M C2 C3 C4 NC C2 C3 C4 NC C2 C3 C4 NC
